# Can the concepts of depression and quality of life be integrated using a time perspective?

**DOI:** 10.1186/1477-7525-3-1

**Published:** 2005-01-05

**Authors:** Margaret Moore, Stefan Höfer, Hannah McGee, Lena Ring

**Affiliations:** 1 Royal College of Surgeons in Ireland, Department of Psychology, Mercer St Lwr, Dublin 2, Ireland; 2 Belfast City Hospital, Department of Health Clinical Psychology, Northern Ireland, UK

**Keywords:** depression, quality of life, Schedule for the Evaluation of Individual Quality of Life, theory, time orientation

## Abstract

**Background:**

Little is understood about the conceptual relationship of depression and quality of life (QoL). Judgments concerning both, implicitly or explicitly, involve a time perspective. The aim of this study was to test de Leval's theoretical model linking depression and QoL with a time perspective. The model predicts that changes in cognitions about one's past, present and future QoL, will be associated with changes in depressive symptomatology.

**Methods:**

Eighteen psychiatric in-patients with a clinically confirmed diagnosis of depression were assessed on commencing treatment and 12 weeks later. QoL was assessed by the Schedule for Evaluation of Individual Quality of Life (SEIQoL), depression by the Beck Depression Inventory (BDI-II) and hopelessness by the Beck Hopelessness Scale (BHS). Time perspective was incorporated by asking QoL questions about the past, present and future.

**Results:**

Depression and hopelessness were associated with a poorer present QoL. Depression lowered present QoL but did not alter future QoL, as these remained consistently high whether participants were depressed or recovering. However, depressed individuals had a larger gap between their actual present QoL and future (aspired to) QoL. Changes in QoL were influenced by depression and hopelessness. Contrary to the model, perception of "past" QoL was not affected by depression or hopelessness.

**Conclusions:**

de Leval's model was largely confirmed. Thus depression and hopelessness influence a person's present and future QoL. The analysis of a temporal horizon was helpful in understanding the link between depression and QoL.

## Background

Assessment of quality of life (QoL) has become increasingly important in health care, particularly as an evaluative method to measure outcomes of the impact of disease and interventions. To date it is unclear how research on QoL relates to other psychological constructs such as depression and anxiety. Many clinical studies assess a number of related psychosocial dimensions but without a theoretical basis for the unique contribution of each. On an intuitive level, QoL and depression can appear as opposing phenomena – crudely representing all the positive and negative aspects of well-being. Poor QoL is sometimes seen as a consequence of depression [1-3]. On the other hand, poor QoL may also be a precursor to depression. In other formulations, depression can be seen as a component of QoL. Whatever the implicit models of their interrelationships, there has been little theoretical attention or research to understand the relationship between depression and QoL.

A theoretical approach developed by de Leval [4,5,8] tries to capture and highlight possible relationships between depression and QoL in a "three-time-dimension" theory. This theory links depression and QoL on a timeline of the past-present-future. Time can be perceived objectively and subjectively. It consists of three dimensions: past, present and future. The presence of psychopathology, e.g. depression has been found to influence time perception. For instance, individuals who are depressed have been reported as finding that time passes more slowly [11]. In comparison to others, depressed individuals also have a temporal focus, which is less future directed and more focused on the past.

The "three-time-dimension" theory describes the dislocated temporal horizon of the depressed patient. It situates both depression and quality of life as part of a continuum in time rather than as independent phenomena. De Leval proposes that, for the depressed individual, time passes slowly, the present is dissociated from the past and the potential for the future is lost or viewed with hopelessness [4]. During the course of their depression, individuals want to go back to their past when things were perceived as better. This searching for the past becomes the individual's future. According to de Leval, depressed individuals have two pasts: the actual past when they were well and the past as a position they wish to regain or aspire to, i.e. the 'therapeutic future', when things were better than they are in the present. In the proposed model by de Leval, in accordance with DSM-III-R criteria, depression is referred to as "ill-being". Ill-being is the current or present state of the patient experiencing depression. De Leval uses the term depression for "phenomenological-depression", i.e., depression as perceived by the individual in question and it is placed on the past-present timeline. She proposes that this "phenomenological-depression" is related to the perception of a gap between a healthy past and a present ill-being. The greater the gap between past and present, the greater the phenomenological-depression.

**Figure 1:**
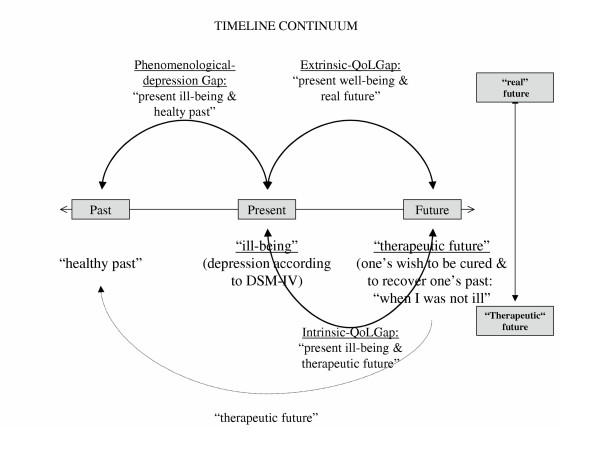
Illustration of de Leval's model

In de Leval's theory, QoL is perceived as the gap between actual experience and future aspirations. Whereas depression is placed on the past-present timeline, QoL is placed along the timeline using present and future. QoL according to this model is defined as being "the appropriateness of future aspirations to the present" or "the making present of the future". The larger the gap, the lower the QoL of the individual (Figure 1). Although not included explicitly in de Leval's theory, the concept of hopelessness as described by Beck [2] is worth considering given its timeline focus on negative evaluations of the future.

In a cross-sectional study, de Leval [5] examined the three-time-dimension theory in a group of 110 clinically depressed psychiatric patients. They completed a 30-item questionnaire – the Three-Time-dimension Synoptic Scale (3TSS – French version) developed by the author [6]. Questions were chosen to reflect the content of existing mental health scales and each question asked about their feelings now, their feelings in the past (before being depressed) and what they wanted to feel in the future. Findings indicated preliminary support for the theory. No other study has to our knowledge tested Leval's theory empirically.

The present study sought to advance the understanding about the conceptual relationship of depression and QoL by empirically testing de Leval's model in a longitudinal study. It also sought to assess the model using a previously developed individualised system for assessing QoL. If de Leval's proposals concerning the centrality of the temporal assessment are correct, then any QoL assessment instrument which measures the present can be adapted to measure the past and the future as aspired to. In this study, the model was operationalised as follows: scores on standardised depression measures were taken to demonstrate an individual's level of depression, i.e., 'present ill-being'. QoL was the gap between an individual's present status ('*present ill-being*' in the case of depression) and anticipated status (i.e '*therapeutic future*') and was measured by the discrepancy between *present *and *future *actual QoL scores. The level of *phenomenological depression *– i.e. the gap between a person's perceived *past *and *present *"*ill-being*" was measured by the discrepancy between the past and present actual QoL scores. These gaps are referred to as time comparisons gaps. The full model is displayed in Figure 1. In addition to components of de Leval's model, aspirational QoL was measured and assessed for all three time periods: past, present and future. This was included to provide further information about the gap between where an individual is and where he/she would like to be. These gaps are referred to as preference comparisons gaps. It was hypothesized that the greater the gap between actual and aspirational scores at any time (past/present/future), the worse the QoL and vice versa. This reflects Calmans definition of QoL. He defines QoL as the gap between actual QoL and preferred QoL [3]. As an additional measure of an individual's perception of the future hopelessness was included . The model was tested at two time points: when individuals were clinically depressed (time 1) and approximately three months later when their progress towards recovery could be estimated (time 2).

The following hypotheses were tested in order to validate de Leval's model and additional components:

Hypothesis 1: The size of the gap between actual and past QoL scores is larger when patients are depressed, in comparison to when they are less/not depressed.

Hypothesis 1a: The therapeutic future is the view of a healthy past. Therefore scores for past and future QoL are equal.

Hypothesis 2: The change in depression scores (present "ill-being") from time 1 to time 2 predicts the change in the gap between *actual *and *aspirational *QoL scores.

Hypothesis 3: The change in hopelessness scores from time 1 to time 2 predicts the change in the gap between *actual *and *aspirational *QoL scores.

Hypothesis 4: A reduction in depression narrows the gap between the past and present, and enhances QoL by narrowing the gap between present and future QoL.

## Methods

Consecutive psychiatric in-patients (N = 27) were approached to participate in a longitudinal study of depression. Study inclusion criteria were age ≥ 18 years, diagnosis of moderate to severe uni-polar depression (DSM-IV-criteria), a moderate or severe depression rating on the Beck Depression Inventory II [1], no cognitive disabilities (Mini Mental State Exam, [7]). Exclusion criteria were diagnosis of severe psychopathology or borderline personality disorder, more than two previous episodes of depression that were of more than three months duration and history of prolonged substance abuse or use of psychotropic medication, which would impede participation in a research interview. Patients were recruited as soon after admission as was deemed appropriate by medical staff.

### Instruments

#### Quality of life (QoL)

QoL was assessed by the Schedule for the Evaluation of Individual Quality of Life (SEIQoL) [12], a well-established method of assessing QoL which incorporates the value system of the individual participant. To reduce interview demands for depressed participants, the shorter direct weighting (SEIQoL-DW) procedure was applied [9]. In the first step of the standard SEIQoL procedure, participants are asked to nominate the five areas of their life (cues) that are most important to them. In the second step, participants rate their current status or level of functioning on each cue. Ratings are against a vertical axis anchored from "worst possibly" to "best possibly".

The final assessment step in SEIQoL involves quantifying the relative importance (weight) of each cue to the judgment of participants' overall QoL. This is obtained by using a weighting disk, consisting of five interlocking coloured disks that can be rotated around a central point to form a pie chart. These disks are labelled with the five nominated cues and are adjusted by participants until they are satisfied that the proportion of the pie chart displayed by each life area accurately reflects the relative importance they attach to these life areas. The SEIQoL Index score is calculated by multiplying each cue weight with the relevant level of functioning. These five scores then are summed. The scale of the SEIQoL Index score ranges from worst possible (0) to best possible (100). This was done for each time anchor and point.

#### Time perspective

To measure the temporal nature of the gap in terms of perceptions of their actual and aspirational past, present and future QoL according to the Leval's theory and Calman's definition of QoL, participants were asked to rate the status of each nominated SEIQoL cue (i.e. how were you doing?) based on the following questions:

Past:

- Where were you before you got depressed? (actual past)

- Where would you have liked to have been? (aspirational past)

Present: 

- Where are you now? (actual present)

- Where would you like to be? (aspirational present)

Future:

- Where do you expect to be in a year’s time? (actual future)

- Where would you like to be in a year’s time? (aspirational future)

Thus scores were taken for actual present, aspirational present, actual past, aspirational past and actual future, aspirational future.

#### Depression

The Beck Depression Inventory (BDI), a 21 item self-report instrument for measuring the severity of depression in adults, was developed for the assessment of symptoms corresponding to criteria for diagnosing depressive disorders based on the DSM-IV. In this study an updated version is used (BDI-II) [1]. The BDI-II requires about 10 minutes to complete and has a two week time-frame including today. Items are rated on a four point scale ranging from 0 to 3, with higher scores reflecting more severe depression. A cut-off score of 20 was used. The psychometric properties have been well established [1].

#### Hopelessness

The Hopelessness Scale [2] is a 20 item self-report instrument designed to assess pessimistic expectations. Items are rated true (1) or false (0), with a higher score indicating hopelessness. Internal consistency (split-half reliability) exceeds .90 in a range of samples and concurrent validity has been established [2].

## Results

Of 27 patients approached, 24 gave written consent and took part in the study (89% response rate). Over the three month follow-up period, six patients dropped out (75% follow-up rate). Information on the complete sample of 18 patients was analysed. Sociodemographic and clinical details are presented in table 1.

**Table 1:** Sociodemographic and clinical profile of recruited sample (N = 24)

	Mean ± SD	%	Min	Max
Gender (% male)		50		
Age (years)	36.7 ± 14.4		18	72
Marital status				
Married/partner		42		
Single		54		
Separated		4		
Occupation:				
Employed		50		
Unemployed/retired		16		
Student		21		
Homemaker		13		
Residence (% urban)		71		
Time since hospital admission (days)	5 ± 2.7		1	9
No. of previous episodes of depression	0.7 ± 0.7		0	2
Duration of previous episodes of depression (weeks)	2.6 ± 3.6		<1	12

Baseline scores of the depression measure (BDI-II) showed high levels of severity, which were reduced significantly after three months (p < .001, table 2). While at baseline all 18 patients scored above 20, only six patients remained above this threshold at three months follow-up. The mean hopelessness score (BHS) of 10 at baseline dropped significantly by 5 (p < .002).

**Table 2:** Profile of mental health at baseline and three month follow-up (N = 18)

	Baseline		three months			
	Mean	SD	Mean	SD	t	p

Depression						
BDI-II	34.0	9.5	15.0	11.4	4.8	<.001
Hopelessness						
BHS	10.0	5.8	5.0	4.9	3.2	.002

The five most commonly mentioned cues for the SEIQoL at time 1 were: mental health, family of origin, work marriage/relationship, friends and leisure. There were no new cues introduced at time 2. This is consistent with previous research on cue profiles across varying populations. The actual present level of overall QoL was very low at baseline (time 1, see table 3). This increased significantly over three months. Aspirational level of present QoL (where one would like to be now) was significantly higher at both baseline and three months then the actual present level. QoL did not change significantly over time (p = .102).

**Table 3:** Quality of life (QoL) over time and actual and aspirational "gaps" at baseline and three months measured by SEIQoL Index Score

		Actual	t	p	Aspirational	t	p	t	p
Present	Baseline	35.4 ± 19.1			88.2 ± 11.4			10.5	<.001
			3.2	.002		1.3	.102		
	three months	61.1 ± 22.8			88.5 ± 8.5			5.5	<.001
									
Past	Baseline	70.2 ± 13.7			87.0 ± 10.5			4.8	<.001
			.2	.411		1.5	.07		
	three months	68.7 ± 18.8			83.7 ± 8.4			4.4	<.001
									
Future	Baseline	66.8 ± 21.0			90.8 ± 10.5			5.1	<.001
			.3	.362		.2	.396		
	three months	74.4 ± 23.6			91.5 ± 7.5			3.6	.001

Actual past level of overall QoL did not change from baseline to three months later. Aspirational past QoL (where one would have liked to be then) was significantly higher at baseline and three months than actual past and this did not change over time (table 3). Actual future QoL did not change significantly over the three months follow-up. Actual future QoL was significantly lower at baseline and three months than the aspirational future QoL. Aspirational future QoL was consistently high over time.

**Figure 2:**
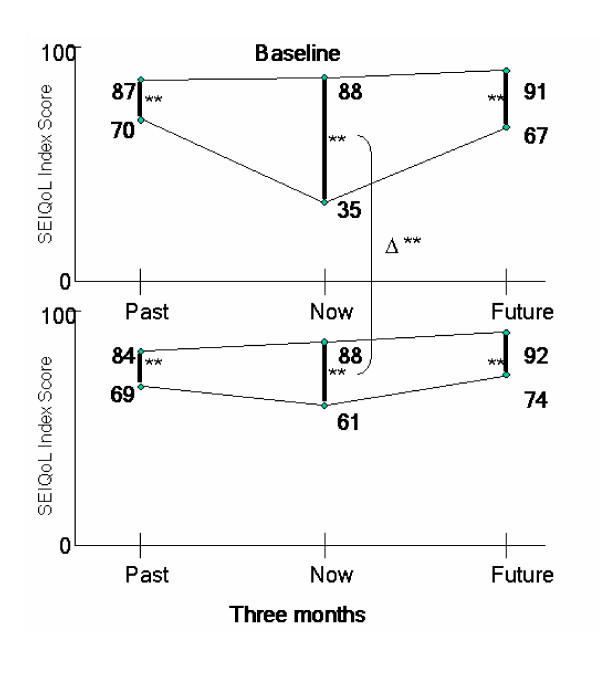
Illustration of the preference comparison gaps (actual/aspirational QoL) for the three time points (past, present and future) at baseline and three months. Δ represents the change from baseline to three months for the time anchor "present" ** p < .001

The gap between actual and aspirational QoL is presented in table 4. The biggest gap was perceived between baseline actual present level of QoL and aspirational present QoL. This large gap was significantly reduced after three months. This confirms hypothesis 1. Other gaps remained unchanged (figure 2). Correlations between the preference comparison QoL gaps for present, past and future assessments with the severity of depression (d) showed significant high correlations for the present (p) and future (f) gap at baseline (1) and three months (2) (r_pd1 _= .468, r_fd1 _= .475, both p < .05, r_pd2 _= .815, r_fd2 _= .730, both p < .01). In addition, correlations between the QoL preference comparison gaps for present, past and future assessment and hopelessness (h) were statistically significant at baseline for the present (p) gap and at three months (2) for present (p) and future (f) gaps (r_ph1 _= .567, r_ph2 _= .586, r_fh2 _= .422, all p < .05). There was no difference between actual past and actual future QoL scores at baseline and three months (p = .475, p = .528). This confirms hypothesis 1a.

**Table 4:** Differences between actual and aspirational QoL "gaps" at baseline and three months as measured by the SEIQoL Index Score

Time	Baseline actual/aspirational gap (M ± SD)	Three months actual/aspirational gap (M ± SD)	F	Time
Present	52.7 ± 21.0	27.6 ± 21.2	9.21	.007
Past	16.8 ± 14.7	14.9 ± 14.9	.154	.699
Future	23.9 ± 19.6	17.0 ± 17.9	2.86	.109

Multiple regression was conducted to investigate whether depression and hopelessness contribute to the change in the size of the gap of actual and aspirational QoL over time. Changes in depression and hopelessness scores over time were entered as independent variables in a multiple regression, while the change in gap scores was entered as the dependent variable. A significant amount of variance was explained by changes in the present and future gaps for actual and aspirational QoL (52.4%, 59,9% respectively both p < 0.01). Change in depression contributed to change (reduction) in the gap between present actual and aspirational Qol (β =.676). Change in hopelessness (β =.589) was the significant contributing factor for change (reduction) in the gap between actual and aspirational future QoL (table 5). This confirms hypothesis 2 and 3 in parts.

**Table 5:** Multiple regression analyses to explain the change in the actual/aspirational gap by change in Beck Depression Inventory (BDI) and Beck Hopelessness Scale (BHS) change scores from time 1 to time 2.

Δ in actual/aspirational Gap	Variable name^1^	R^2^	p-value	β	p
Present		.524	.004		
	Δ BDI_12_			.676	.020
	Δ BHS_12_			.063	.813
Past		.043	.720		
	Δ BDI_12_			.053	.888
	Δ BHS_12_			.165	.661
Future		.599	.001		
	Δ BDI_12_			.230	.352
	Δ BHS_12_			.589	.026

^1^Change of Beck Depression Inventory (BDI) and change of Beck Hopelessness Scale (BHS) were entered as predicting variables in these analyses

**Figure 3:**
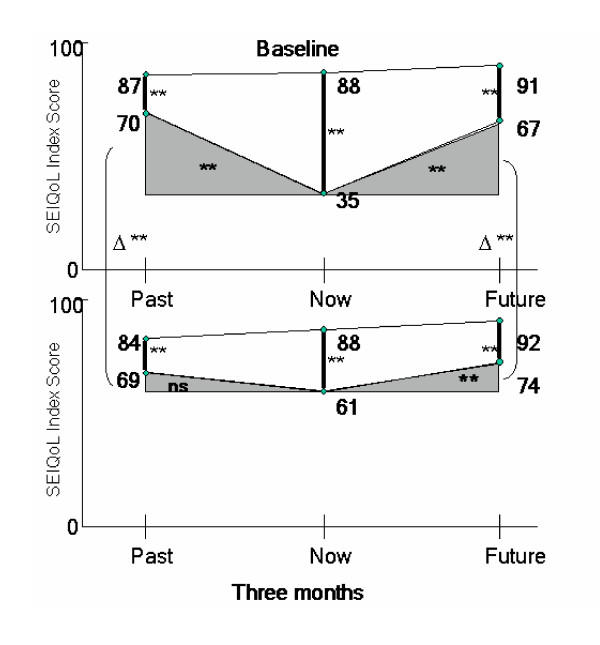
Illustration of the time comparison gaps (past-present/present-future QoL; shaded areas) for the three time points (past, present and future) at baseline and three months. Δ represents the change from baseline to three months for the time anchor "past" and "future" ** p < .001; ns ... not significant

In table 6, the differences of present and past (phenomenological-depression) and present and future QoL (quality of life) are assessed according to de Leval's model. As already reported, baseline actual present QoL scores were considerably lower than actual past scores (35.4 ± 19.1 vs. 70.2 ± 13.7, p <.001). This significant gap (34.8 ± 22.5) between present and past QoL was significantly reduced over three months to a gap of 7.6 ± 30.9 (p = .011). At the three month follow-up, there was no statistical difference in the gap between actual present and past QoL perception (figure 3). The gap between present and future QoL was large and significant at baseline (31.3 ± 27.2, p < .001). Differences remained significant at the 3 month follow-up (13.3 ± 13.5, p = .001) with higher QoL levels for the future. However, the reduction in the gap over time was also significant (p = .010).

**Table 6:** Testing de Leval's model: time gap comparison for actual QoL scores measured by SEIQoL Index Score

	QoL at Baseline	t	p	QoL at three months	t	p
Past	70.2 ± 13.7			68.7 ± 18.8		
		6.5	p < .001		1.0	.310
Present	35.4 ± 19.1			61.1 ± 22.8		
Present	35.4 ± 19.1			61.1 ± 22.8		
		4.8	p < .001		4.2	.001
Future	66.8 ± 21.0			74.4 ± 23.6		

To investigate how depression and hopelessness may influence the change in the size of the gaps proposed by de Leval, multiple regression analysis was conducted. Change in depression and hopelessness scores over time were entered as independent variables in a multiple regression, while the change in the gap over time, was entered as the dependent variable. Changes in the size of the two time comparison gaps (past/present; present/future) were not influenced by change in depression or hopelessness scores (R^2 ^= .281, p = .09; R^2 ^= .162, p = .266). This partly disconfirms hypothesis 4

## Discussion

In this study the relationship of QoL and depression was investigated in two ways. First the preference comparison gaps were assessed, i.e. where patients perceive themselves to be and where they wish to be. This was done for all three time dimensions (past, present and future). Secondly, the time comparison gap was assessed, i.e. where patients see themselves now in comparison to the past, and in comparison to the future. Both approaches were carried out on the basis of de Leval's model linking depression to all three time dimensions.

Our findings are consistent with previous results, showing that people use their current affective state as a basis for making judgments of how happy and satisfied they are with their lives. A depressed person will usually see his or her well-being, social functioning and living conditions as worse than they appear to an independent observer or to patients themselves after recovery – the so called "affective fallacy" [10]. Clinically depressed patients were assessed at two time points before and three months after admission to a psychiatric hospital. Clinical measures (depression and hopelessness) showed significant improvements for all 18 patients, indicating successful treatment. Alongside the reduction in depression, actual present QoL improved significantly over time. In contrast, actual past QoL did not change over time. Successful treatment of depression had no influence of the individual's perception of the past. In addition, there was no significant change in actual future QoL, i.e. how patients realistically estimated their QoL to be one year from now. Thus assessments of the past and of the expected future did not change when patients recovered from a depressive episode. Aspirational QoL was the same between the three time dimensions and remained constant over time. There was always a preference comparisons gap over time – independent of depression or recovering depression.

Our findings suggest the larger the present preference comparison gap, the greater the depression. While the preference comparison gap of the past did not change over time with the decrease of depression, there was a trend for the future preference comparison gap that might become statistically significant with a larger sample size. However, aspirations were not affected by depression, since they remained the same for past, present and future from baseline in hospital to three months later. Overall these findings may indicate that a reduction in the present preference gap should be the main goal for therapeutic actions for depressed patients. An achievable target may be to keep the same preference comparison gap between and over time points to maintain a healthy homeostasis and a good QoL (figure 2). Interestingly, the mean level of aspirational QoL was not at the top of the possible scale (100). This may reflect an individual "QoL set-point" as discussed by Seligman [14]. He proposes that an individual might be genetically predetermined, to a particular set-point, to which he/she might return after both ups and downs. In therapy then the goal might be to help an individual actively work to return to his/her set point as soon as possible.

Successful treatment of depression, indicated by a significant change of BDI-II and BHS scores, was accompanied by a significant reduction in the gap between present actual and aspirational QoL. A reduction in the gap between actual present QoL with the aspirational QoL ("where one would like to be right here and now") reflects a more positive view of the present and is accompanied by significant improvements in QoL. Depression was consistently correlated with the time comparison gap between present and future QoL assessments. However, depression was more highly associated with the present and future preference comparison gap at three months. This suggests, that depression may be more highly associated with the actual/aspirational gap when people are less depressed. In contrast, hopelessness was consistently correlated with the preference comparison future gaps and only at baseline with the gap of present actual/aspirational QoL. The gap between the actual past and aspirational past QoL was not explained by either depression or hopelessness. Change in depression or hopelessness was important in reducing the preference comparison gap for present and future QoL assessments. Change in depression was important in reducing the size of the present gap. Change in hopelessness significantly contributed to reducing the size of the future gap. Given that present "ill-being" is seen as depression, while hopelessness is future orientated and directed, this finding makes sense. This has implications for therapy: to improve the QoL of patients with depression, relief from depression must be provided. Hopelessness needs to be tackled in addition to achieving a realistic future QoL perspective.

To a large extend this study confirms de Leval's theory. The assessment of de Leval's model: present and future QoL (quality of life) and past and present (phenomenological-depression) showed interesting results. The theoretical time comparison gap between a "healthy past" and present "ill-being" was confirmed by the significant difference between patients' perceptions of actual present and future QoL, at baseline. Over time, following appropriate treatment, this time comparison gap should close and QoL should improve, if the treatment is considered to be successful. The significant reduction of the past-present gap over time, and the loss in significance in size of this gap three months later, further confirms de Leval's model. This reduction of the time comparison gap between the present "ill-being" and a "healthy past" resulted in an improvement in QoL. The reduction was achieved by a change in the actual view of the present, leaving the view of the past unchanged (figure 3).

According to de Leval's model, recovery from depression is achieved when the appraisal of present and past QoL are very close, i.e. a very narrow gap. This means that a reduction of the gap between present and future QoL should improve current QoL. Although this study showed a decrease in depression over time by standard measures of depression and the size of the time comparison gap was significantly reduced between the two time points, the size of the gap did remain significant between the present and future at three months follow up. This finding may either indicate that the patients were still depressed at three months, or this part of the model could not be confirmed (figure 3). de Leval argues that therapeutic future is the view of a healthy past. Therefore one would expect similar scores for past and future QoL. This was supported by our findings for both time points. The change in the size of the time comparison gaps was not predicted by the change in either hopelessness or depression scores over time. This suggests that the change in time comparison gap's may be influenced by other variables, not assessed in this study. Depression lowered a person's actual QoL, but not their aspirational QoL. This was consistent whether a person was depressed or improving and for each of the time dimensions (past-present-future). The recollection of the past, either actual or aspirational did not change for the depressed or recovering person, neither did their view of the future. Thus for this patient group, the perception of the past and the future was not altered by depression status, which might be linked to individual QoL set points.

### Limitations

Since we tested the model in a small and specialised sample, the findings are not generalisable. However, our study did test de Leval's model in a clinically depressed sample which is the focus of the most likely benefit of understanding the interplay of the two phenomena of depression and quality of life. It is also a challenging group to recruit, engage with and maintain in a research project.

## Conclusion

This study showed that depression influenced individual QoL by lowering the person's actual QoL. Thus depression was associated with a larger gap between current reality (actual perceptions) and patient aspirations and realistic expectations. Aspirations for past, present and future QoL remained the same over time. However, patients actual appraisal of their present QoL improved with successful treatment of depression, reflected by a closure of the present preference gap.

de Leval's model was largely confirmed. Thus depression and hopelessness influence a person's present and future QoL. The analysis of a temporal horizon was very helpful in understanding the link between depression and QoL. Therapeutic interventions can be considered as closing the gap between a person's present QoL and their present aspirations and realistic expectations for the future. This could be achieved, according to the data presented here, by changing the view of the present and not necessarily by changing the level of expected future QoL. Knowing the persons aspirational QoL level may be a helpful guide for the therapist in that it provides important information about a depressed person's position in the progress towards what he or she considers to be recovery.

## Authors' contributions

Margaret Moore contributed to conception and design, acquisition, analysis and interpretation of the data and revised the article. Stefan Höfer drafted the article and contributed to the analysis and interpretation of the data. Hannah McGee contributed to the conception and design and interpretation of the data and revised the article. Lena Ring contributed to the interpretation and revised the article.

All authors have given final approval of the version to be published
